# Fiber reinforced composite supported restoration of congenitally missing tooth by minimally invasive approach: Two years follow-up

**DOI:** 10.12669/pjms.37.3.3873

**Published:** 2021

**Authors:** Muhammad Qasim Javed

**Affiliations:** 1 Muhammad Qasim Javed, FCPS, Assistant Professor, Department of Conservative Dental Sciences and Endodontics, College of Dentistry, Qassim University, Buraydah, Qassim, Saudi Arabia

**Keywords:** Hypodontia, Fiber-reinforced Composite, Fixed partial dentures

## Abstract

Maxillary lateral incisor is the most frequent congenitally missing anterior tooth of the permanent dentition. The absence of the anterior tooth can adversely affect the production/transmission of speech sounds, mental health, and facial aesthetics of an individual. Considering this, prosthetic rehabilitation of missing front tooth is important. The treatment alternatives include implant supported single crown, conventional fixed partial dentures (FPDs), and resin bonded FPDs that are unilaterally or bilaterally supported by metallic wings. However, with the development in adhesive dentistry fiber reinforced composite (FRC) supported FPDs have provided a workable substitute for traditional techniques because of their improved esthetics, minimal invasiveness, less cost, enhanced bond strength, and revocable nature. The current case, reports the two years follow up of twenty-four years old female patient, for whom the congenitally absent maxillary right lateral incisor was restored with FRC supported FPD.

## INTRODUCTION

Tooth agenesis (Hypodontia) is the most commonly observed craniofacial developmental anomaly in humans. The prevalence of tooth agenesis varies in different populations and ranges between 1.6% and 6.9%. The majority of subjects affected by tooth agenesis have either one or two missing teeth; with the maxillary lateral incisors and the permanent second premolars as the most frequently missing teeth.^1^ The missing front teeth can affect the phonetics, facial aesthetics and psychological wellbeing of the patient. Therefore, it is important to restore the missing anterior teeth.^2^ The treatment options include conventional fixed partial dentures (FPDs), implant supported restoration and resin bonded FPDs (Maryland Bridge) with unilateral or bilateral metal anchorage.^3^ The factors to be considered while replacing the missing teeth include cost, aesthetics and minimal invasion. Considering this, the conventional FPDs are most invasive that involve the circumferential and occlusal reduction of adjacent teeth.^2^ Also, the provision of Implant supported restoration at times is not possible due to time constraints, financial constraints, deficient hard and soft tissue.^3^ Although the resin bonded FPDs are a minimally invasive approach but their utilization is limited in the anterior region as a result of unaesthetic appearance of metallic wings and weak tooth metal bond.^4^ Conversely, fiber reinforced composite (FRC) supported FPDs have enhanced esthetics, low cost, better bond strength, and can be fabricated by both direct and indirect methods with minimal tooth preparation.^4^ Hence, the FRC supported FPDs offer a viable alternative treatment modality.^4^

The present case report describes the two years follow up of 24 years old female patient with congenitally missing right maxillary lateral incisor. The missing tooth was restored with FRC supported FPD.

## CASE REPORT

**Fig.1:**
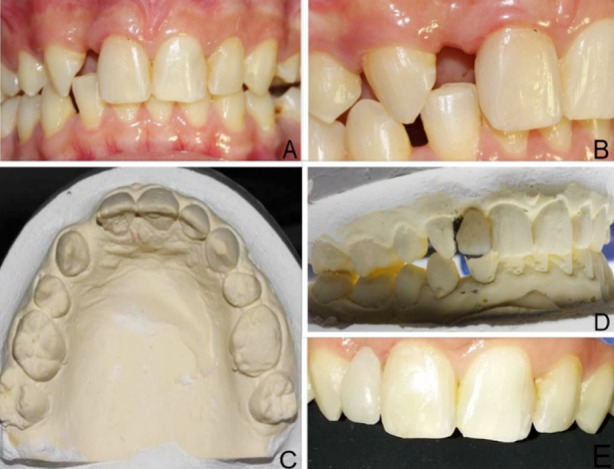
**(A)** Preoperative frontal view **(B)** Preoperative lateral view **(C)** Model showing missing teeth **(D)** Working models with pontic tooth **(E)** Frontal view at the time of cementation

Twenty-four years old systemically fit female presented to the Department of Operative Dentistry, Riphah International University, Pakistan, with the chief complaint of missing maxillary right lateral incisor tooth (Tooth#12) (Fig.1A). The history revealed that patient had retained tooth#52 that was extracted a year ago. The findings of extra-oral examination were insignificant. The intraoral examination showed that tooth#12 was missing, mesio-distal space for tooth#12 was limited (6mm) because of mesial shifting of right canine, buccal bone was insufficient in the area of Tooth#12, and all second premolars were missing (Fig.1B and 1C). The radiographic examination confirmed the diagnosis of hypodontia. The patient’s oral hygiene and periodontal status was good. Moreover, the potential abutment teeth were without any restoration and overjet was with in normal limits. The treatment options were discussed with the patient along with their pros, cons and prognosis. The patient opted for FRC reinforced FPD.

**Fig.2:**
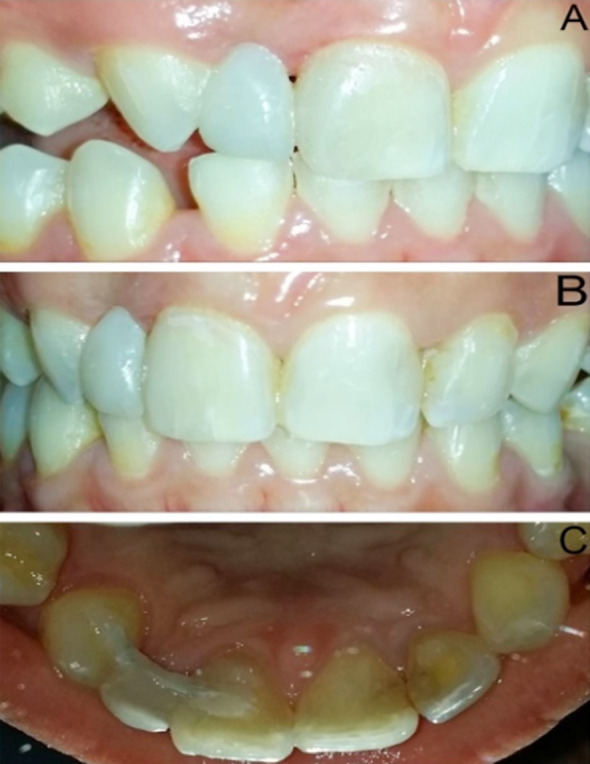
**(A)** Occlusal check in dynamic occlusion **(B)** Frontal view **(C)** Palatal view at 2 years follow up

The shade of patient’s teeth was established in the daylight. The impressions of both maxillary and mandibular arches were taken and model was poured for freehand fabrication of the modified ridge lap pontic by utilizing layering technique and micro-hybrid composites (3M-Filtek™ Z250 Universal Restorative) (Fig.1D). The intertwined glass fibers impregnated with light-cured composite resin (Interlig, Angelus, Brazil) was used for the pontic support. The length of FRC (Interlig, Angelus, Brazil) was adjusted by utilizing dental floss on the working model. The channels of 1.5 mm depth and 3mm width were grooved on the palatal surfaces of pontic and abutment teeth by utilizing diamond bur (DI-S41, Mani, Japan). The grooved area of pontic was air abraded with Al_2_O_3_ particles and then coated with organic silane. After 60 seconds the silane was air dried and adhesion process was initiated. The palatal surfaces of abutment teeth were etched with 37% phosphoric acid (Vivadent N-etch) and bonding agent (Adper single bond, 3M, USA) was applied on the abutments where the FRC would bond. The bonding agent was cured for 20 seconds by LED curing light (Woodpecker, China). Subsequently, the pontic’s grooved area was coated with bonding agent, light cured and attached to adjacent teeth by using flowable composite (Filtek Z350 Flowable-3M,USA). Later the flowable composite was applied to the palatal groove on pontic and FRC was fixed and cured (Fig.1E). This was followed by the application of microhybrid composite (3M-Filtek™ Z250) and 40 seconds of curing for fixing the FRC to pontic and abutment teeth. Lastly, after finishing and polishing of the composite, occlusal adjustments were done in static and dynamic occlusion (Fig.2A). The patient was given oral hygiene advice and instructed not to bite on anterior teeth. The six monthly periodic follow up was conducted. The restoration was found to have satisfactory esthetics with slight color change and was functional without fracture and debonding at two years follow up (Fig.2B and 2C).

## DISCUSSION

The congenitally missing anterior tooth can have negative impact on the psychosocial well-being and smile of the person.^5^ The cost, time available, aesthetics, invasiveness and tooth preservation are the factors that influence the restoration of missing tooth.^2^ In the present case, patient opted for FRC supported FPD because of minimally invasive esthetic nature of the treatment, her time and financial constraints. Also, FRC supported FPD is easy to apply, repairable and have better bond strength as compare metal-winged Maryland Bridge.^4^

Interlig was used as it was comprised of glass fibers that were pre-impregnated with light curable resin system which after polymerization transforms into semi-interpenetrating polymer network. This network provides better adhesion for the composite material with the FRC framework with better durability and bond strength.^6^ Micro-hybrid composites with separate enamel and dentin shades were used for the fabrication of pontic, which offered the aesthetically acceptable outcome for the anterior tooth.^7^ The modified ridge lap pontic design enhanced its cleansability, polishability and provided an appropriate emergence profile of pontic with smooth convex surfaces.^2^,^8^ The indirect method of fabrication was preferred as it offered improved working conditions, increased degree of composite polymerization, ease of finishing and polishing in comparison to direct method.^4^ The 1.5 mm deep grooves were prepared in the abutment teeth in order to create a space for placing FRC.^9^ Air abrasion of the pontic and silane treatment was done to improve the wetting by resin adhesive.^10^

The revolution in restorative dental procedures has been the inevitable consequence of advancements in adhesive dentistry^11^. As a result, the FRC supported FPD were considered as a viable short-term restorative option mainly because of the reservations on the longevity of restorations, however, current systematic review has suggested them as the medium-term alternative for managing the missing single anterior tooth.^4^ Also the revocable and the minimally invasive nature of the treatment means that alternative treatment can be applied whenever the patient have time and financial resources.^12^
